# Correction of upper eyelid retraction following ptosis surgery using Hering’s law: A case report

**DOI:** 10.1097/MD.0000000000041624

**Published:** 2025-02-28

**Authors:** Runhui Pang, Zhaochuan Liu, Huixing Wang, Juan Wang, Junhu Shi, Li Xiao, Ping Bai

**Affiliations:** a Department of Ocular Plastics, Hebei Eye Hospital, Hebei Provincial Key Laboratory of Ophthalmology, Hebei Provincial Clinical Research Center for Eye Diseases, Hebei Eye Hospital, Xingtai, Hebei, China; b Department of Ophthalmology, Beijing Tongren Hospital, Beijing Ophthalmology and Visual Science Key Laboratory, Capital Medical University, Beijing, China.

**Keywords:** blepharoptosis, eyelid retraction, Hering’s law

## Abstract

**Rationale:**

Blepharoptosis is a common disorder characterized by abnormal eyelid position that affects both visual function and esthetics. Postoperative upper eyelid retraction, which is often due to overcorrection, is a major problem. Under normal circumstances, the operation is usually performed on the overcorrected eye. Here, we present the case of a patient who had previously undergone surgery to correct right upper blepharoplasty, which resulted in asymmetry. Utilizing Hering’s law, we decided to correct only the left eye, resulting in remarkable bilateral symmetry after surgery. This proves that for patients with ptosis after upper eyelid surgery, a comprehensive approach should be considered rather than just one treatment method.

**Patient concerns:**

Here, we report a case of overcorrection of ptosis in a male patient who underwent corrective surgery.

**Diagnoses:**

Based on the patient’s ocular examination, appearance, medical history, measurement of levator muscle strength, and MRD1 measurement, the diagnosis was postoperative overcorrection of ptosis of the right upper eyelid.

**Interventions:**

In this case, preoperative examination revealed a reduction in the size of the right eye when the left eyelid was lifted, which was in accordance with Hering’s law. Consequently, surgical intervention was limited to the left eye, resulting in excellent bilateral symmetry after surgery.

**Outcomes:**

The surgery was successful and resulted in symmetry between both eyes. We followed up with the patient for a year and he was extremely satisfied with the surgical results. The outpatient follow-up revealed no discomfort.

**Lessons:**

Postoperative upper eyelid retraction following monocular ptosis correction is a rare condition. The conventional treatment approach typically involves reoperation on the affected eye. However, considering the importance of Hering’s law in the management of ptosis, excellent outcomes were achieved in this case through surgery on the contralateral, unaffected eye. When treating a patient, the operation should be tailored to their specific condition.

## 1. Introduction

Congenital blepharoptosis is a common eye disorder that affects not only the aesthetics but also the physical, psychological and the visual function of patients.^[1,2]^ Surgical intervention is often necessary, with postoperative asymmetry requiring corrective measures. Regardless of whether the ptosis is unilateral or bilateral, it is important to pay attention to Hering’s law preoperatively and postoperatively. Varying degrees of severity of ptosis can result in one eyelid appearing normal while the other droops.^[3–6]^ Correction of the drooping eyelid may subsequently affect the contralateral eyelid. The patient we were dealing with was a unique case in which we found on examination that after surgical correction of the normal eyelid, the contralateral retracted eyelid returned to its normal position. Therefore, we are presenting this particular case for reporting purposes.

## 2. Case presentation

The patient, male, 25 years old, presented with a history of difficulties opening his right eye since childhood. He underwent surgery 10 years ago to shorten the right upper eyelid muscle, which resulted in persistent asymmetry of the right upper eyelid. On admission, visual acuity was found to be 20/20 in the right eye and 20/20 in the left eye. A scar was visible 6 mm from the lid margin of the right eye, with the upper eyelid 2 mm above the cornea and exposed sclera. The left eye covered the cornea about 4 mm. The strength of the levator muscle was 11 mm in the right eye and 9 mm in the left eye. The marginal reflex distance 1 was 2 mm in the right eye and 1 mm in the left eye. There was no restriction of movement in any direction for either eye. Upper eyelid retraction following the ptosis surgery presents a challenge in achieving symmetrical eyelid position and function. In this case, the patient desired correction of upper eyelid retraction in the right eye due to its relatively larger size. However, the preoperative assessment according to the principles of Hering’s law revealed an asymmetric response to the elevation test. During the elevation test, with the patient staring straight ahead, manual elevation of the left eyelid above the corneal limbus resulted in a marked decrease in the position of the right eyelid (Fig. 1). Consequently, the decision was made to perform an upper blepharoplasty on the left eye. This case highlights the importance of using Hering’s law-based assessments for surgical planning and optimizing the outcome of upper eyelid retraction.

**Figure 1.**
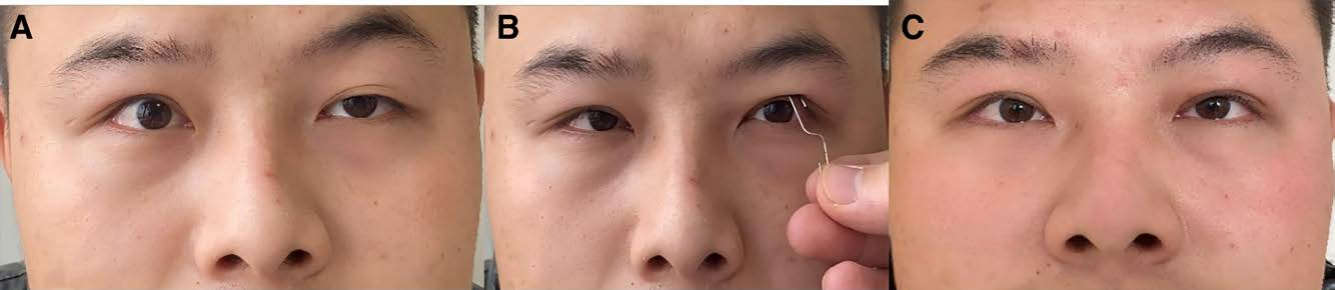
(A) Preoperative photo, (B) applying Hering’s law for preoperative examination, and (C) 1 year postoperative.

### 2.1. Surgical procedure

The patient underwent reconstructive surgery for symmetry of the upper eyelid. He entered the operating room and sterile drapes were applied. A 6 mm wide upper eyelid crease was marked on the left eye and local anesthesia with 2% lidocaine was administered. The skin was incised along the marked line and the lower part of the orbicularis oculi muscle was removed. The levator aponeurosis and Müller muscle were exposed and a composite flap was formed by separating them approximately 20 mm upwards. The 15 mm composite flap was attached to the tarsus of the upper eyelid with absorbable 5-0 sutures. Excess tissue and protruding orbital fat was removed and the orbital septum was sutured. The upper lid margin was positioned 2 mm below the cornea and the excess skin was removed. The skin was then sutured with 5-0 silk sutures in the style of a double eyelid. Postoperative follow-up: 6 months after surgery, the patient was followed up and was found to have symmetrical upper eyelid creases, both eyelids exhibit symmetrical redundant skin folds with normal curvature, without entropion or ectropion. The height of the eyelid fissures is consistent bilaterally, with no recession. There were no signs of eyelid malformation or recurrence, and the marginal reflex distance 1 was 2 mm in both eyes and did not regress (Fig. 2).

**Figure 2.**
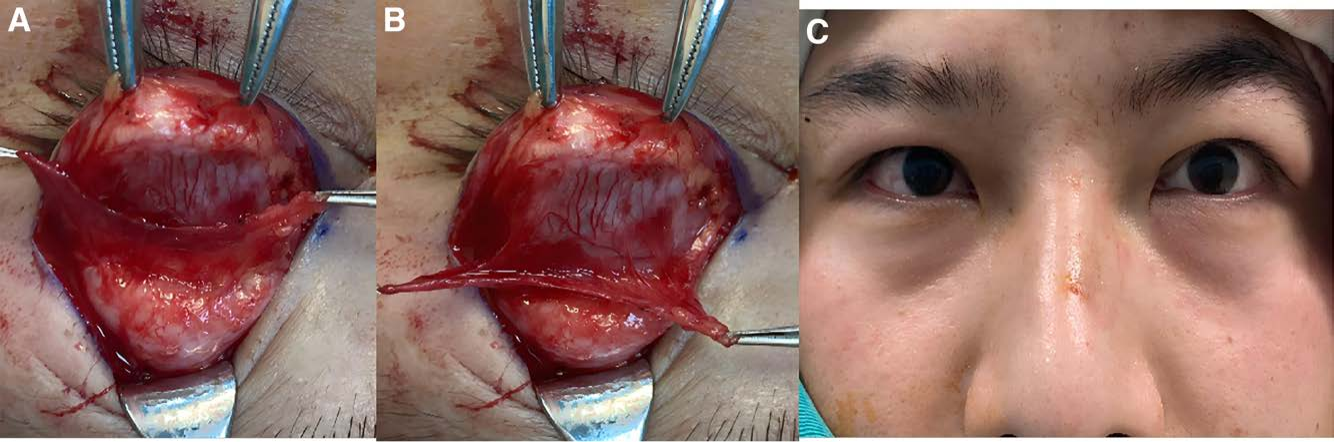
(A) Intraoperative frontal view of the levator muscle. (B) Intraoperative posterior view of the levator muscle. (C) Intraoperative photo.

## 3. Discussion

Congenital ptosis is a prevalent ocular malformation characterized by upper eyelid drooping that obscures part or all of the pupil, leading to partial or complete visual field loss. The upper eyelid margin typically overlaps the upper corneal margin by more than 2 mm during primary gaze, indicating compromised function of the levator palpebrae superioris muscle.^[7]^ Studies report a prevalence of congenital ptosis ranging from 4.7% to 13.5% in the adult population, with an observed increase in incidence with age.^[8,9]^ Surgical intervention is often necessary for congenital ptosis, with the choice of procedure depending on the severity of ptosis and associated factors. Traditionally, the eye with the more severe ptosis is addressed if both eyes are affected.^[10]^ Established surgical techniques include frontalis sling surgery and levator muscle shortening.^[10,11]^ However, during preoperative assessment, alongside evaluating levator muscle strength, marginal reflex distance, and ocular motility, careful consideration of Hering’s law is crucial for achieving optimal surgical outcomes.^[12]^

Hering’s law, originally proposed to explain coordinated binocular movements through neural innervation of the extraocular muscles, was suggested by Walsh in 1957 to potentially apply to eyelid movements as well. He observed a compensatory retraction of the contralateral upper eyelid in unilateral ptosis, a phenomenon attributable to Hering’s law. In the treatment of congenital ptosis, the eye with the more severe ptosis is usually operated on if both eyes have different degrees of ptosis. Currently recognized surgical procedures include frontalis sling surgery and shortening of the levator muscle.^[11]^ In addition to evaluating levator muscle strength, marginal reflex distance, and ocular motility during ptosis examination, Hering’s law should also be emphasized.^[12]^

A study of 60 patients with upper eyelid retraction following ptosis surgery evaluated the effectiveness of corrective surgery using composite tissue flaps. The procedure involved creating composite tissue flaps by separating the pretarsal tissue below the upper lid margin and the Müller muscle with the conjunctiva or tarsus. The lower edge of the tissue flap was then sutured to the upper lid margin to reduce the upper lid retraction. Satisfactory results were achieved in 61 of the 71 eyelids (86%), with mild ptosis observed in 6 cases (8%) and recurrence of eyelid retraction in 4 cases (6%). All unsuccessful cases were successfully resolved by early reoperation for further correction of ptosis or retraction.^[13]^ Techniques for correcting eyelid retraction include mullerectomy, levator recession, levator lengthening, and spacer grafting.^[14]^ For severe retractions requiring significant lengthening, grafting may be necessary using materials such as collagen sheeting, cadaver skin, or fascia lata.^[15]^ Notably, all reported surgeries focus on the affected eye, with no documented cases of intervention on the normal eye. Another study^[3]^ reported that after unilateral ptosis correction, the contralateral eyelid height decreases on average by 0.2 to 0.8 mm, with a decrease of more than 1 mm in 17% of patients. Additionally, 5% of patients required surgical revision within the first postoperative year. However, all revisions were performed on the originally operated eye, with no reported surgical intervention on the contralateral eye.

Hering’s law, recently gaining attention, can be evaluated through elevation, cover, and phenylephrine tests. The elevation test is considered the most sensitive method for detecting contralateral pseudo-elevation. However, the optimal duration for maintaining manual elevation remains undetermined, with opinions ranging from a few seconds^[16]^ to 30 seconds.^[17]^ While less sensitive than the elevation test, the cover test typically involves covering the drooping eyelid for approximately 15 seconds.^[18]^

In this case, we performed the elevation test for a duration exceeding 30 seconds. Based on the preoperative examination results, we shortened the levator muscle, performing surgery solely on the left eye. Postoperatively, excellent symmetry was achieved between both eyes, highlighting the importance of Hering’s law in ptosis correction. The levator function test is not only useful for correcting or ruling out a preoperative pseudo-elevation of the contralateral eyelid position but also plays a crucial role in selecting the surgical eye when postoperative asymmetry occurs. If the contralateral eyelid has normal muscle strength and position, surgery should focus on correcting the retracted eyelid to prevent postoperative eyelid retraction. However, if the contralateral eyelid exhibits ptosis with a positive levator function test, surgery should be performed on the contralateral eyelid.

In this case we observed, the patient experienced secondary eyelid retraction in the right eye following a previous operation to correct ptosis. According to standard treatment protocols, the right eye should have undergone repositioning or lengthening of the levator muscle. However, during preoperative examination, we discovered that when the left lid margin was repositioned to its normal position, the right lid margin was immediately displaced downwards. Based on these findings, we decided to perform surgery to shorten the levator muscle in the left eye while leaving the right eye untreated. Postoperatively, the symmetry of both eyes was excellent, clearly demonstrating that Hering’s law plays a decisive role not only in the correction of ptosis but also in cases of under- or overcorrection after surgery.

Additionally, an increase in upper eyelid width was observed postoperatively in the right eye. Although these findings are limited to a single case, The limitation of this study is that it involves a single patient, and the limited sample size constrains our findings. In future practice, we will examine similar patients more closely to provide better and more appropriate treatment options. They suggest overcorrection after ptosis surgery may not always necessitate operating on the initially treated eye. Further studies with larger patient populations are warranted to confirm these observations and establish more generalizable surgical decision-making guidelines based on Hering’s law principles.

## Author contributions

**Writing – original draft:** Runhui Pang

**Data curation:** Huixing Wang, Junhu Shi, Li Xiao.

**Investigation:** Juan Wang.

**Writing – review & editing:** Zhaochuan Liu, Ping Bai.
